# Bioresorbable Scaffolds: Contemporary Status and Future Directions

**DOI:** 10.3389/fcvm.2020.589571

**Published:** 2020-11-30

**Authors:** Xiang Peng, Wenbo Qu, Ying Jia, Yani Wang, Bo Yu, Jinwei Tian

**Affiliations:** ^1^Department of Cardiology, The Second Affiliated Hospital of Harbin Medical University, Harbin, China; ^2^The Key Laboratory of Myocardial Ischemia, Harbin Medical University, Ministry of Education, Harbin, China; ^3^Guangxi Key Laboratory of Diabetic Systems Medicine, Guilin Medical University, Guilin, China

**Keywords:** bioresorbable scaffolds, percutaneous coronary intervention, absorb bioresorbable vascular scaffold, scaffold thrombosis, intravascular imaging

## Abstract

Percutaneous coronary intervention, which is safe, effective, and timely, has become an important treatment for coronary artery diseases and has been widely used in clinical practice. However, there are still some problems that urgently need to be solved. Permanent vessel caging through metallic implants not only prevents the process of positive vessel remodeling and the restoration of vascular physiology but also makes the future revascularization of target vessels more difficult. Bioresorbable scaffolds (BRSs) have been developed as a potential solution to avoid the above adverse reactions caused by permanent metallic devices. BRSs provide temporary support to the vessel wall in the short term and then gradually degrade over time to restore the natural state of coronary arteries. Nonetheless, long-term follow-up of large-scale trials has drawn considerable attention to the safety of BRSs, and the significantly increased risk of late scaffold thrombosis (ScT) limits its clinical application. In this review, we summarize the current status and clinical experiences of BRSs to understand the application prospects and limitations of these devices. In addition, we focus on ScT after implantation, as it is currently the primary drawback of BRS. We also analyze the causes of ScT and discuss improvements required to overcome this serious drawback and to move the field forward.

## Introduction

Over the past four decades, percutaneous coronary intervention (PCI), as a landmark breakthrough in cardiology, has revolutionized the treatment of coronary artery disease. To improve the prognosis of patients with coronary heart disease, new instruments in the field of coronary intervention are constantly being developed and improved. The incidence of restenosis is as high as 20–50% after treatment with percutaneous transluminal coronary angioplasty (PTCA) and bare metal stents (BMSs). By carrying antiproliferative drugs, drug-eluting stents (DES) reduce the restenosis rate to 5% (1), but to date, problems such as delayed endothelialization and local inflammatory reaction remain and may lead to neoatherosclerosis and late stent thrombosis (ST). With the rise of the concept of “vascular restoration” for coronary heart disease intervention, bioresorbable scaffolds (BRSs) have emerged. In an ideal state, BRSs can provide temporary mechanical support to the vessel wall and ensure revascularization at an early stage, avoiding vessel collapse and negative reconstruction. The subsequent degradation and complete resorption of scaffolds can leave only the native vessel to preserve the physiological integrity, which not only avoids the permanent residue of metallic struts but also enables coronary adaptive positive remodeling and normal reactive vasomotion. In this review, we summarize the current trial outcomes and clinical evidence for BRSs and discuss the factors that may be associated with the increased risk of scaffold thrombosis (ScT), including patient and lesion characteristics, the premature design of BRSs, the poor application of optimal implantation technique and intravascular imaging, and the disruption of dual antiplatelet therapy (DAPT). We hope to reduce the risk of adverse events and improve the long-term prognosis of patients by optimizing these aspects.

## Current Status of BRSs

Compared with DES, the most prominent feature of BRSs is bioresorbability. Therefore, the core requirement of BRS manufacturing is that absorbable materials have both high biocompatibility and excellent flexibility as well as mechanical properties, which can avoid scaffold fractures during stent implantation and allow minor elastic shrinkage in later stages at the same time. Importantly, the process of degradation in BRSs needs to be consistent with the healing cycle of coronary artery lesions. Thus, a BRS needs to provide effective support to the vascular wall for at least 6 months after implantation (2). Driven by the above demands, bioabsorbable polymers or metals have become two major trends in the selection of BRS materials. Table 1 summarizes the principal characteristics of the currently available and upcoming BRSs.

**Table 1:** Features of current and upcoming BRSs.

	**Device**	**Manufacturer**	**CE mark (year)**	**Backbone**	**Coating**	**Strut Thickness** **(μm)**	**Antiproliferative drug**	**Drug dose**	**Resorption time (months)**
Biodegradable polymer scaffold	Absorb BVS	Abbott vascular	2012	PLLA	PDLLA	157	Everolimus	100 μg/mm^2^	24–48
	DESolve Nx	Elixir medical	2014	PLLA	Polylactide based polymer	150	Novolimus	5 μg/mm	24
	DESolve Cx	Elixir medical	2017	PLLA	Polylactide based polymer	120	Novolimus	5 μg/mm	24
	Fantom	REVA medical	2017	PTD-PC	PTD-PC	125	Sirolimus	115 μg (3.0 ×18 mm)	36
	ART 18AZ	Arterial remodeling technology	2015	PDLLA	N/A	170	None	None	6
	Mirage	Manli cardiology		PLLA fibers	PLLA	125	Sirolimus	N/A	14
	FAST	Boston scientific		PLLA	N/A	≤99	Everolimus	N/A	12–24
	MeRes-100	Meril life science		PLLA	PDLLA	100	Sirolimus	1.25 μg/mm^2^	24
	FORTITUDE	Amaranth medical		PLLA	PDLLA	150	Sirolimus	96 mg/cm^2^	12–24
	APTITUDE	Amaranth medical		PLLA	PDLLA	120	Sirolimus	96 mg/cm^2^	>36
	MAGNITUDE	Amaranth medical		PLLA	PDLLA	98	Sirolimus	96 mg/cm^2^	24–36
	Xinsorb	HuaAn biotechnology		PLLA	PDLLA	160	Sirolimus	12 μg/mm	24–36
	Firesorb	MicroPort medical		PLLA	PDLLA	100-125	Sirolimus	4 μg/mm	36
	NeoVas	Lepu medical		PLLA	PDLLA	170	Sirolimus	15.3 μg/mm	36
	IDEAL biostent	Xenogenics		PAE salicylic acid	Adipic acid	200	Sirolimus	N/A	6–9
Bioabsorbable metal scaffold	DREAMS 1G	Biotronik	2015	Magnesium alloy	PLGA	125	Paclitaxel	7.4 μg/cm^2^	9–12
	Magmaris (DREAMS 2G)	Biotronik	2016	Magnesium alloy	PLLA	150	Sirolimus	140 mg/cm^2^	9–12
	IBS	Life tech scientific		Iron alloy	PDLLA	70	Sirolimus	235 μg/cm^2^	12–24

*BVS, bioresorbable vascular scaffold; IBS, iron bioresorbable scaffold; N/A, not available; PDLLA, poly-d,l-lactide; PLLA, poly-l-lactide; PTD-PC=desaminotyrosine polycarbonate; PLGA, poly-lactic-co-glycolic acid*.

### Bioabsorbable Polymer Scaffolds

The backbone of biodegradable polymer scaffolds is mainly composed of poly-l-lactide (PLLA) monopolymer. PLLA is a kind of semicrystalline polymer that usually requires 2–3 years to be almost completely resorbed *in vivo*. Through the tricarboxylic acid cycle, PLLA is ultimately degraded into water and carbon dioxide, leading to the complete bioresorption of the implanted materials. However, compared with traditional stent materials, such as steel or cobalt-chromium (CoCr), PLLA has poorer mechanical properties such as stiffness and ductility, especially the elastic modulus closely related to radial strength, which is 66 times lower than that of CoCr (3). To have a similar capacity to metallic stents to support the vessel, scaffolds prepared from PLLA tend to have thicker scaffolds to compensate for the reduced tensile and radial strength. At present, international companies and scientific research institutions have developed a variety of biodegradable polymer scaffolds, such as Absorb, DESolve, and Fantom.

#### Absorb BVS

The Absorb Bioresorbable Vascular Scaffold (Absorb BVS; Abbott Vascular) is the first drug-eluting BRS that received a CE marking for clinical use. Absorb BVS is composed of a PLLA-derived polymer backbone with 157 μm strut thickness, coated with a layer of a completely absorbable and release rate-controllable mixture (containing poly-d, l-lactide (PDLLA) and everolimus). The antiproliferation drug is 80% released at 30 days, and the polymer is almost completely resorbed by 2 years. An overview of the clinical trials with Absorb BVS is presented in Table 2.

**Table 2:** Clinical trials with Absorb BVS.

**Study** **(Identifiers)**	**Aim**	**Start date**	**Follow-up** **(BVS, *n*)**	**Death (%)**	**Cardiac death (%)**	**MI (%)**	**ID-TLR** **(%)**	**TLF** **(%)**	**D/P ST (%)**
ABSORB cohort A (NCT00300131)	The First in Man clinical study to evaluate the feasibility and safety of Absorb BVS in patients with single de novo native coronary artery lesions	Mar 2006	5 years (29)	6.9	0	3.4	0	3.4	0
ABSORB Cohort B (NCT00856856)	To evaluate the safety and performance of the Absorb BVS in patients with a maximum of two de novo native coronary artery lesions located in two different major epicardial vessels.	Mar 2009	5 year s (100)	3.0	0	3.0	8.0	14.0	0
ABSORB extend (NCT01023789)	To evaluate performance of the Absorb BVS in a lesion subset representative of daily clinical practice, including calcified lesions, total occlusions, long lesions, and small vessels.	Jan 2010	3 years (812)	N/A	2.1	4.0	3.1	9.2	2.2
ABSORB II (NCT01425281)	To compare the safety, efficacy and performance of Absorb BVS Against XIENCE EES in patients with de novo native coronary artery lesions	Nov 2011	3 years (335)	2.5	0.9	8.3	6.2	10.5	2.8
ASSURE (NCT01583608)	To evaluate the safety, performance and efficacy of the Absorb BVS in patients with de novo native coronary artery lesions in a real-world setting.	Apr 2012	1 year (183)	1.1	0.5	1.7	N/A	5.0	0
EVERBIO II (NCT01711931)	To compare the efficacy and safety of everolimus- and biolimus-bluting stents with Absorb BVS.	Oct 2012	2 years (78)	2.6	1.3	5.1	14.1	20.5	1.2
ABSORB III (NCT01751906)	To evaluate the safety and effectiveness of the Absorb BVS System compared to the XIENCE EES	Dec 2012	5 years (1,322)	7.0	2.7	12.7	9.5	17.5	2.5
PRAGUE-19 (ISRCTN43696201)	To evaluate the safety and effectiveness of the Absorb BVS in patients with STEMI.	Dec 2012	5 years (79)	6.3	3.8	2.5	3.8	12.6	2.5
ABSORB Japan (NCT01844284)	To evaluate the safety and effectiveness of Absorb BVS in Japanese population with de novo native coronary artery lesions compared with XIENCE EES	Apr 2013	5 years (254)	11.8	7.9	7.5	8.3	11.0	3.8
ABSORB China (NCT01923740)	To evaluate the safety and efficacy of the Absorb BVS compared to the XIENCE EES in patients with up to two de novo native coronary artery lesions in separate epicardial vessels.	Jul 2013	3 years (236)	0.8	0.4	3.4	4.2	6.8	0.9
AIDA (NCT01858077)	To evaluate the efficacy and performance of Absorb BVS versus XIENCE EES in an all-comers contemporary population with coronary lesions.	Aug 2013	2 years (924)	3.5	2.0	7.1	3.0	10.3	3.5
ISAR- ABSORB MI (NCT01942070)	To evaluate the clinical performance of Absorb BVS versus EES in patients undergoing PCI in the setting of acute MI.	Sep 2013	1 years (173)	3.5	2.3	1.8	4.8	7.0	1.8
GABI-R (NCT02066623)	To evaluate the safety and performance of the ABSORB BVS in patients with coronary artery stenosis	Nov 2013	2 years (2,709)	2.9	0.8	5.0	N/A	6.7	2.8
TROFI II (NCT01986803)	To assess the neointimal healing score of Absorb BVS versus XIENCE EES in patients with STEMI.	Jan 2014	3 years (95)	2.1	2.1	3.2	3.2	5.3	2.1
ABSORB IV (NCT02173379)	To continue evaluate the safety and effectiveness as well as the potential short and long-term benefits of Absorb BVS compared to XIENCE EES	July 2014	1 year (1,296)	1.2	0.8	6.2	2.9	7.6	0.7

*BVS, bioresorbable vascular scaffold; D/P ST, definite or probable stent thrombosis; EES, everolimus eluting coronary stent; ID-TLR, ischemia driven-target-lesion revascularization; MI, myocardial infarction; N/A, not available; STEMI, ST-elevation myocardial infarction; TLF, target lesion failure*.

The ABSORB I trial is the first-in-human study to assess the safety and efficacy of Absorb BVS at 5 years of follow-up. Scaffold thrombosis was not observed in any patient, and the occurrence of major adverse cardiac effects (MACE) was 3.4 and 11% in cohorts A and B, respectively (4, 5). Optical coherence tomography (OCT) investigation showed that the formation of a neointima layer resembled a thick fibrous cap during the vascular healing process, which perhaps suggested that Absorb BVS may contribute to the formation of stable endothelium (6). At the same time, a study included a subset of patients from the ABSORB Cohort A and B trials aiming to assess endothelial-dependent or independent artery vasomotion at 12 and 24 months. The vasodilatory response to acetylcholine was quantitatively associated with a reduction in polymeric strut echogenicity over time and a small number of necrotic core areas, which indicated the feasibility of restoring artery vasomotion as the BRS degrades (7). These findings demonstrate that the advantages of Absorb BVS are that they can preserve the normal integrity and physiology of the treated segment, which is impossible to observe after metallic DES implantation.

However, with the accumulation of clinical evidence, a series of studies suggest that the rates of target lesion failure (TLF, a composite of cardiac death, myocardial infarction, and target lesion revascularization) and ScT with the Absorb BVS are significantly higher than those with the DES. A large-scale meta-analysis of seven randomized ABSORB trials including 5,583 patients assigned to receive treatment with an Absorb BVS (*n* = 3,261) or a cobalt-chromium everolimus-eluting stent (CoCr-EES) (*n* = 2,322) showed that the relative risks of the device-oriented composite endpoint and thrombosis at 2 years with the Absorb BVS were higher than those with the CoCr-EES (9.4% vs. 7.4%, *p* = 0.0059, 2.3% vs. 0.7%, *p* < 0.0001, respectively). Furthermore, higher rates of target vessel-related myocardial infarction (MI) (5.8% vs. 3.2%, *p* = 0.0003) and ischemia-driven target lesion revascularization (TLR) (5.3% vs. 3.9%, *p* = 0.0090) were reported in the Absorb BVS groups, with similar levels of cardiac mortality in both groups (8). An individual-patient-data pooled meta-analysis, in which 2,164 patients received Absorb BVS and 1,225 received CoCr-EES, reported similar results. Compared with the CoCr-EES, the Absorb BVS was associated with significantly higher incidences of TLF (11.7% vs. 8.1%, *p* = 0.006) and thrombosis (2.4% vs. 0.6%, *p* = 0.001) during 3 years of follow-up. Moreover, the risks of TLF and thrombosis between 1 and 3 years were higher with the Absorb BVS as well (6.1% vs. 3.9%, *p* = 0.02, and 1.1% vs. 0.0%, *p* < 0.0001, respectively) (9).

One recently reported trend is that the end of the period of excess risk is consistent with the time point of complete resorption of Absorb BVS within ~3 years, and there seems to be no sign of an excess of all patient- and device-oriented endpoints for the Absorb BVS over the CoCr-EES between 3 and 5 years. In the ABSORB III trial, though the cumulative incidence of TLF through 5 years remained numerically increased with the Absorb BVS compared with the CoCr-EES (23.2% vs. 19.9, *p* = 0.07) due to the higher rates of adverse events in the first 3 years, a substantial reduction in the relative hazards of the Absorb BVS for TLF (hazard ratio [HR] 0.83 vs. 1.35, *p* = 0.052) and ScT (HR 0.26 vs. 3.23, *p* = 0.056) was reported between 3 and 5 years after implantation (10). Similarly, 5 year clinical outcomes from the ABSORB Japan trial indicated no significant differences in any patient- and device-oriented adverse outcome between the Absorb BVS and CoCr-EES throughout the 5 years or between 3 and 5 years following resorption of the scaffold (11). A pooled meta-analysis including four trials of Absorb BVS vs. CoCr-EES with 3,384 patients showed that the increased risks of TLF and device thrombosis between 3 and 5 years post-procedure were markedly lower than those in the first 3 years (4.3% vs. 4.5%, HR 0.92, 95% confidence interval [CI] 0.64 to 1.31, and 0.1% vs. 0.3%, HR 0.44, 95% CI 0.07 to 2.70, respectively). Spline modeling showed that the relative hazard gradually decreased over time after the peak point for the Absorb BVS risk at 2 years after implantation, and the Absorb BVS performed comparably to the CoCr DES with regard to adverse events between 3 and 5 years (12). These longer-term data have rekindled our expectation for the Absorb BVS. By improving stent design and deployment techniques, we may be able to reduce the risk at an early stage and improve the long-term value proposition of the Absorb BVS.

#### DESolve

The DESolve (Elixir Medical) is a PLLA-derived polymeric scaffold with a strut thickness of 150 μm and novolimus coating at a concentration of 5 μg/mm. The polymer reabsorption time is approximately 24 months, and the loss of initial molecular weight is more than 95% in the first 12 months. The main characteristic of this device is that, unlike the Absorb BVS, it has great ductility and self-expansion properties. High flexibility provides tolerance to excessive stretching without risk of scaffold fractures. The unique self-expansion property can help recover the post-deployment diameter and avoid the occurrence of malapposition.

The DESolve First-in-Man trial, which was a prospective multicenter study enrolling 16 patients with *de novo* lesions, demonstrated the feasibility and efficacy of the device. No scaffold thrombosis or device oriented major adverse cardiac events (MACE, including cardiac death, target vessel MI, and clinically indicated TLR) occurred for 12 months. The results of the 6 month follow-up showed low in-scaffold late lumen loss (0.19 ± 0.19 mm by quantitative coronary angiography; QCA) and uniform, thin neointimal coverage (0.12 ± 0.04 mm by OCT), with no scaffold recoil or late malapposition (13). In 2016, data were presented from a prospective, multicenter, non-randomized DESolve Nx trial, which enrolled 126 eligible patients. The rates of MACE and TLR were 7.4% (*n* = 9) and 4.1% (*n* = 5) at 24 months, respectively, including a probable thrombosis within the first month index procedure. QCA evaluation demonstrated that in-scaffold late lumen loss (0.20 ± 0.32 mm) was comparable to currently available stents, and intravascular ultrasonography (IVUS) analysis showed an added benefit for vessel (4.04±0.54 vs. 4.16 ± 0.52, *p* = 0.002), lumen (2.74 ± 0.27 vs. 2.86 ± 0.31, *p* = 0.001) and scaffold (2.75 ± 0.27 vs. 2.94 ± 0.32, *p* < 0.001) dimension enlargement between the post-procedure time and 6 months after implantation, which were earlier than other PLLA-based scaffolds (14). A post marketing clinical study indicated that the cumulative 12 month rates of device-oriented composite endpoints, TLR and ScT were 3.0% (*n* = 3), 3.0% (*n* = 3), and 1.0% (*n* = 1), respectively (15). A study retrospectively compared the performance of the DESolve vs. Absorb BVS, which included 63 Absorb BVS treatments in 35 patients and 50 well-matched DESolve treatments in 37 patients. In the DESolve group, there were larger maximal and minimal scaffold diameters and lower residual in-scaffold area stenosis, with no significant difference in mean and minimal lumen area. Moreover, compared with Absorb BVS, DESolve was more uncommon because of its unique expansion properties (16). Currently, DESolve Cx, a newer version of the DESolve, has a strut thickness of 120 μm, a length of 14–28 mm and a diameter of 2.5–4.0 mm. The first-in-man registry is being evaluated for the safety and effectiveness of DESolve Cx among ~150 patients (17). Of note, there are no clinical trials to compare to the safety profile of DES to date, and a definitive answer on the safety and effectiveness of DESolve cannot be derived.

#### Fantom

The Fantom (Reva Medical) bioresorbable, sirolimus-eluting scaffold is prepared from a desaminotyrosine polycarbonate-based backbone, with a strut thickness of 125 μm. The Fantom scaffolds have an excellent radial strength and the capacity to achieve a single-step continuous inflation. In addition, due to the presence of iodinated tyrosine analog polymer, the characteristic of radiopacity enables the device to deploy more expediently and precisely throughout the whole implantation process, and non-invasive radiologic evaluation of scaffold degradation can be performed *in vivo*. The polymer is fully resorbed by 36 months, with <20% of the molecular mass remaining after the first 12 months. Strut tissue coverage was almost complete (98.1%) as early as 6 months after stent implantation.

The multicenter FANTOM II study evaluated 240 patients implanted with the Fantom scaffolds, and the 6 month clinical outcome showed no deaths, one ScT (0.9%), two MIs (1.7%) and two clinically driven TLRs (1.7%); one patient had both an MI and TLR. The risk of MACE within 6 months occurred in 2.6% of patients; by contrast, other CE-marked BRSs have higher MACE risks, ranging from 3.0 to 5.0% (18). OCT analysis revealed a favorable healing pattern of the Fantom scaffolds at 6 and 9 months, during which the mean and minimal scaffold areas maintained stability and acute malapposition was effectively resolved, with no late vessel wall detachment (19).

### Bioabsorbable Metal Scaffolds

Compared with bioabsorbable polymer scaffolds, bioabsorbable metal scaffolds have higher radial strength and collapse pressure, reduced shortening, and negligible early elastic recoil (<8%). There are two main types of metal materials frequently used for scaffolds at present, including magnesium alloy and iron alloy.

#### Bioabsorbable Magnesium-Based Scaffolds

Magnesium has good mechanical properties and can provide a strength-to-weight ratio that is comparable to that of stainless steel, and another virtue of magnesium-based scaffolds is the substantial resistance to platelet adhesion and aggregation owing to its electrochemical properties (20).

AMS-1 (Biotronik) is the first generation of bioabsorbable magnesium-based scaffolds, with a strut thickness of 165 μm but no layer of antiproliferative drug elution. Although AMS-1 has great mechanical properties and histocompatibility, the results of the PROGRESS-AMS study are unsatisfactory. Owing to the rapid degradation of metal scaffolds, the radial strength was insufficient to support coronary lesions at an early stage, and neointima hyperplasia and negative vascular remodeling were also observed. At 12 months of follow-up, the incidences of restenosis and ischemia-driven TLR were as high as 47.5 and 45%, respectively (21).

For this reason, DREAMS 1G (Biotronik), which has a slow degradation rate, was developed and coated with a mixture of poly-lactic-co-glycolic acid (PLGA) and paclitaxel. The first-in-man, prospective, multicenter BIOSOLVE-I trial, which evaluated the long-term performance of DREAMS 1G in 46 patients, reported the occurrence of three TLFs but no cardiac death or ScT at 3 years. In-scaffold late lumen loss assessed by QCA improved from 0.51 ± 0.46 mm (median 0.28 mm) at 12 months to 0.32 ± 0.32 mm (median 0.20 mm) at a median of 28 months after implantation (22).

The current-generation Magmaris scaffold (formerly known as DREAMS 2 G, Biotronik) has radiopaque markers at both ends, which are made from permanent tantalum, to allow visualization of the scaffold throughout implantation. The scaffold is coated with PLLA polymer instead of PLGA polymer, and the antiproliferative drug was converted from paclitaxel to sirolimus at a dose of 140 μg/cm^2^, resulting in better endothelialization and a reduced inflammatory response. In the BIOSOLVE-II trial, a total of 123 patients with *de novo* coronary artery lesions were enrolled. The 3 year follow-up with Magmaris showed a low TLF rate of 6.8% (*n* = 8; including 2 cardiac deaths, 1 target vessel MI, 2 clinically driven TLRs) and no occurrences of probable or definite thrombosis. Under serial angiographic assessment, in-segment late lumen loss (LLL) and diameter stenosis had a slight increase from 12 to 36 months (0.11 ± 0.28 mm, *p* = 0.060, and 3.8 ± 10.1%, *p* = 0.072, respectively) (23). The prospective, observational BIOSOLVE-IV trial is ongoing and is estimated to enroll 2,054 patients with follow-up for 5 years. Twelve month clinical outcomes of the first 400 patients showed that TLF occurred in 17 patients (4.3%), who needed clinically driven TLR exclusively and in whom the incidence of MI was 0.8% (*n* = 3). One definite ScT (0.3%) was observed on postprocedure day 10 owing to a 5 day interruption of DAPT treatment (24). However, DREAM BRS is not ready for clinical use because the safety and effectiveness of this device has not been evaluated in large-scale prospective randomized trials, and the obtainable data are insufficient in the real world at present.

#### Bioabsorbable Iron-Based Scaffolds

Among all BRSs, the iron bioresorbable scaffold (IBS; Lifetech Scientific) has the thinnest scaffold thickness at 53 μm, and it offers similar support to the other metallic scaffolds. Preclinical results suggest no significant difference between the IBS and the CoCr-EES in area stenosis and intimal thickness at 28, 90, and 180 days. No ScT was observed within 180 days after implantation. Tissue coverage of the IBS was more complete compared with that of the CoCr-EES at the 14 day follow-up (25). Interestingly, the IBS shows good device performance comparable to a current mainstream DES. However, the slow degradation of the IBS scaffold *in vivo* is a considerable drawback. Only 2.0±1.8% mass loss occurred at 3 months after implantation, and it took 13 months for full resorption of the IBS scaffold in the rabbit abdominal aorta (26). The time to complete degradation was up to 53 months after IBS implantation in a pig model, in which the corrosion products derived from this device were removed by macrophages (27). Notably, there is no animal model that can completely replicate the complex environment of human lesion vessels with an implantation, and the reabsorption time of the IBS scaffold in human atherosclerotic vessels is not clear; it may even be longer than that in healthy animal models. Therefore, further improvement is needed to optimize the degradation time and develop the IBS into a promising treatment for patients with coronary artery disease.

### The Newer BRS: Development and Testing

New-generation BRSs may obtain better outcomes and development due to the attractive and promising concept that indicates vessels implanted with BRSs will recover to their natural state. To overcome the pre-BRS shortcomings and obtain widespread application, new-generation BRSs use materials that are absorbable and exhibit stronger mechanical properties with the aim of providing thinner struts and smaller crossing profiles. Brief summaries regarding the new-generation BRS are as follows.

#### FORTITUDE

**Figure 1:**
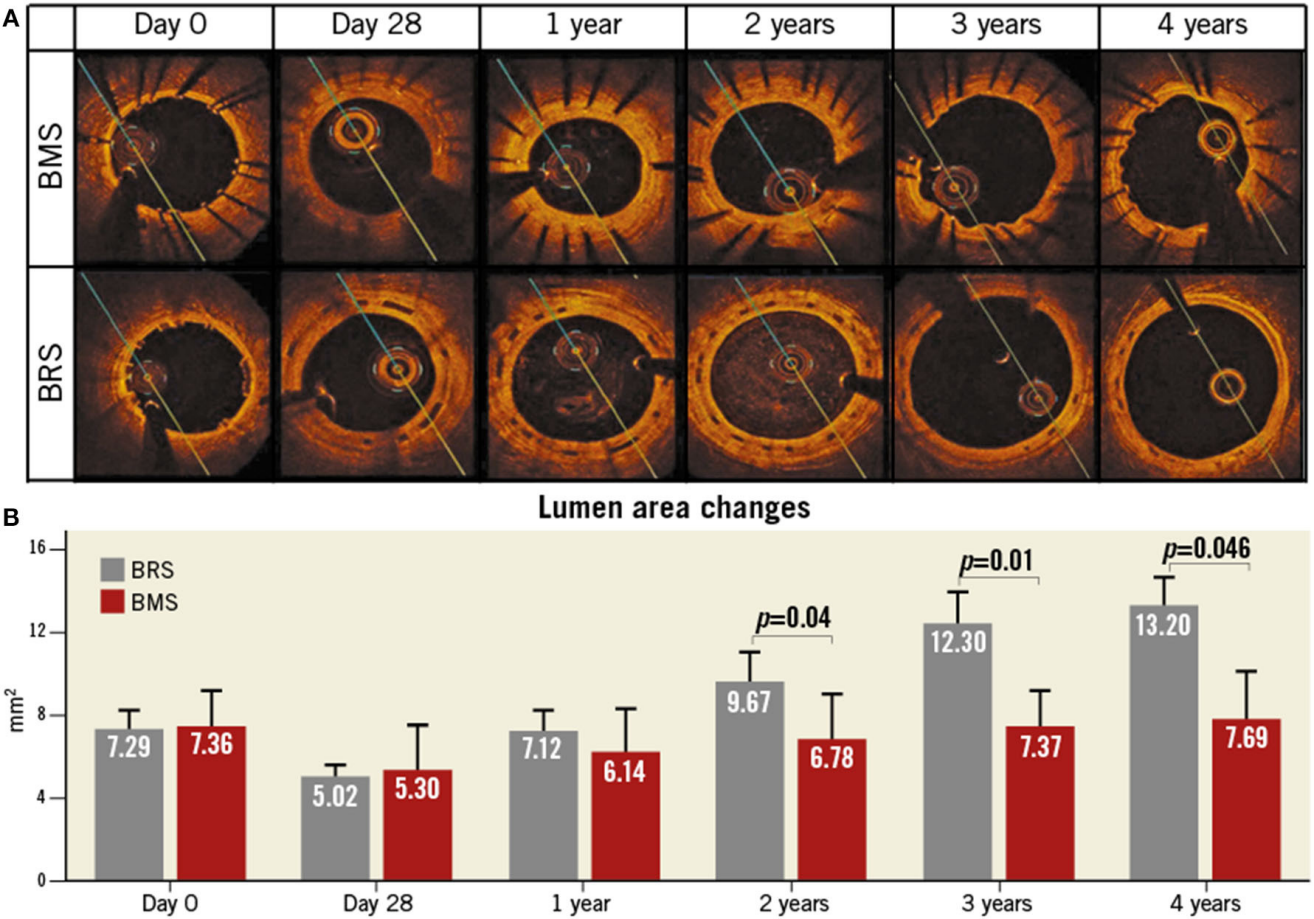
Lumen area changes of BMS and BRS by using OCT to process the longitudinal assessment. **(A)** Typical images to compare the lumen area changes between BMS (top panel) and BRS (bottom panel). **(B)** Quantitative analysis of the relevant in-stent lumen area regarding BMS (red) and BRS (gray) for 4 years. BRS, bioresorbable scaffolds; BMS, bare metal stents; OCT, optical coherence tomography. This Figure is reprinted from ReF.28, Vahl et al. (28), Copyright 2016, with permission from Copyright Clearance Center.

FORTITUDE (Amaranth Medical) consists of a bioresorbable, ultrahigh-molecular-weight and PLLA polymeric scaffold with a PDLLA coating that may release sirolimus and has a strut thickness of 150 μm. The time of complete absorption is ~10 months. To evaluate the healing and the early tissue compatibility in FORTITUDE BRSs, histomorphometric analyses and OCT measurements were performed in 16 adult Yucatan minipigs at 28 days (group 1A) or at 90 days (group 1B). A second group of 4 adult Yucatan minipigs was used for serial OCT measurements at 28 days and at 1, 2, 3, and 4 years (28). Typical images of the lumen area changes of BMS and BRS by using OCT to process the longitudinal assessment are exhibited (Figure 1). That is, FORTITUDE was similar to the control BMS in biocompatibility, safety and efficacy of maintaining coronary arterial patency. However, the arterial patency with BRSs that did not contain antiproliferative drug elution showed evidence of late lumen gain and positive remodeling. A clinical trial enrolling 68 patients showed that the incidence of target vessel failure and ScT is 4.9 and 1.6%, respectively. Moreover, the APTITUDE device, the next generation of scaffolds that is being developed by this company, showed that target vessel failure and ScT did not occur during 24 months in preliminary reports involving 48 patients (28). A multi-center prospective study exhibited the safety and effectiveness of FORTITUDE BRS in patients with low level of target vessel failure and late lumen loss after treating 9 and 24 months (29).

#### XINSORB

XINSORB (HuaAn Biotechnology) is a sirolimus-eluting scaffold that is made of PLLA and has a strut thickness of 160 μm. The efficiency and safety of XINSORB bioresorbable sirolimus-eluting scaffold had been investigated in porcine model, which demonstrated the XINSORB scaffold had an effect on inhibiting neointimal hyperplasia without obvious late device recoil in 180 days follow-up (30). In addition, XINSORB scaffold has similar effects on the acute stent recoil, acute stent malapposition or collapse with metallic stent under a pre-clinical trial using 16 minipigs (31). These pre-clinical trials can provide preliminary data to further studies of XINSORB. A randomized, controlled study with 392 patients, in which 200 randomly received the XINSORB and 192 received the TIVOLI stents (Essen Technology, Beijing), showed that the XINSORB achieved a better outcome in ischemia-driven target lesion revascularization (1.0% vs. 4.9%) and the same outcome for ScT (0.5% vs. 0.5%). Additionally, MACE and ScT were not observed in a trial of XINSORB including 27 cases (32). XINSORB scaffold was effective and safe on treating single *de novo* coronary lesions in a clinical trial including 19 patients implanted XINSORB scaffold with 6 month follow-up, and this trial showed the negative relationship between the healing score and implanted duration or post-dilatation (33).

#### Firesorb

The Firesorb scaffold (MicroPort), which can be absorbed within 36 months, includes a PDLLA abluminal coating that can release sirolimus (4 g/mm) and is composed of PLLA. Its thickness is 125 μm for a device with a diameter of 3.0 mm and 100 μm for a device with a diameter of 2.5 mm. In the first-in-human Future-I study of the Firesorb scaffold that enrolled 45 cases, MI occurred in a patient requiring revascularization at 2 years after implantation, though ScT and cardiac death did not occur (32). In addition, the patients with non-complex coronary lesions exhibited the preliminary effectiveness and feasibility of Firesorb BRS during 3 year follow-up as well (29). The randomized Future- II trial is in progress.

All of these preclinical studies provide a fundamental basis for further clinical studies and application of the newer BRSs for human use. In addition, many clinical studies about newer BRSs are in process, and a specific table is provided to show the ongoing trials with new BRSs (Table 3).

**Table 3:** Ongoing trials with new BRSs.

	**Device**	**Clinical trials identifier**	**Recruitment status**	**Actual study start date**	**Estimated study completion date**	**Study type**	**Enrollment**	**Allocation**	**Masking**
FANTOM II	FANTOM	NCT02539966	Recruiting	Mar-2015	Mar-2023	Interventional	220	Non-randomized	None (open label)
MeReS100-China	MeRes100; XIENCE EES	NCT03454724	Active, not recruiting	Jan-2020	Dec-2024	Interventional	484	Randomized	None (open label)
RENASCENT	FORTITUDE	NCT02255864	Active, not recruiting	Feb-2015	Nov-2020	Interventional	21	N/A	None (open label)
RENASCENT II	APTITUDE	NCT02568462	Active, not recruiting	Nov-2015	Jul-2021	Interventional	60	N/A	None (open label)
FUTURE-I	Firesorb	NCT02659254	Active, not recruiting	Jan-2016	Oct-2021	Interventional	45	N/A	None (open label)
FUTURE-II	Firesorb	NCT02890160	Recruiting	Aug-2017	Oct-2023	Interventional	610	Randomized	Single (participant)
MAGMARIS	Magmaris	NCT03413813	Active, not recruiting	Jul-2017	May-2021	Observational	445	Not applicable	Not applicable
BIOSOLVE-IV	Magmaris	NCT02817802	Recruiting	Aug-2016	Oct-2025	Observational	2054	Not applicable	Not applicable
BESTMAG	Magmaris	NCT03955731	Recruiting	Feb-2019	Feb-2022	Interventional	100	N/A	None (open label)
IBS-FIM	IBS	NCT03509142	Active, not recruiting	Apr-2018	Dec-2024	Interventional	65	N/A	None (open label)

*EES, everolimus-eluting stent; IBS, iron bioresorbable scaffold; N/A, not available*.

## Scaffold Thrombosis

The initial expectation is that BRS will gradually degrade over time, and vasoreactivity will be restored, which can avoid complications caused by permanent metal stent implantation and minimize the risk of late ST. However, with additional long-term follow-up data from large-scale, randomized trials being reported, concerns about the safety of BRS have been raised. At present, ScT is the main ongoing limitation of the current-generation BRS.

**Figure 2:**
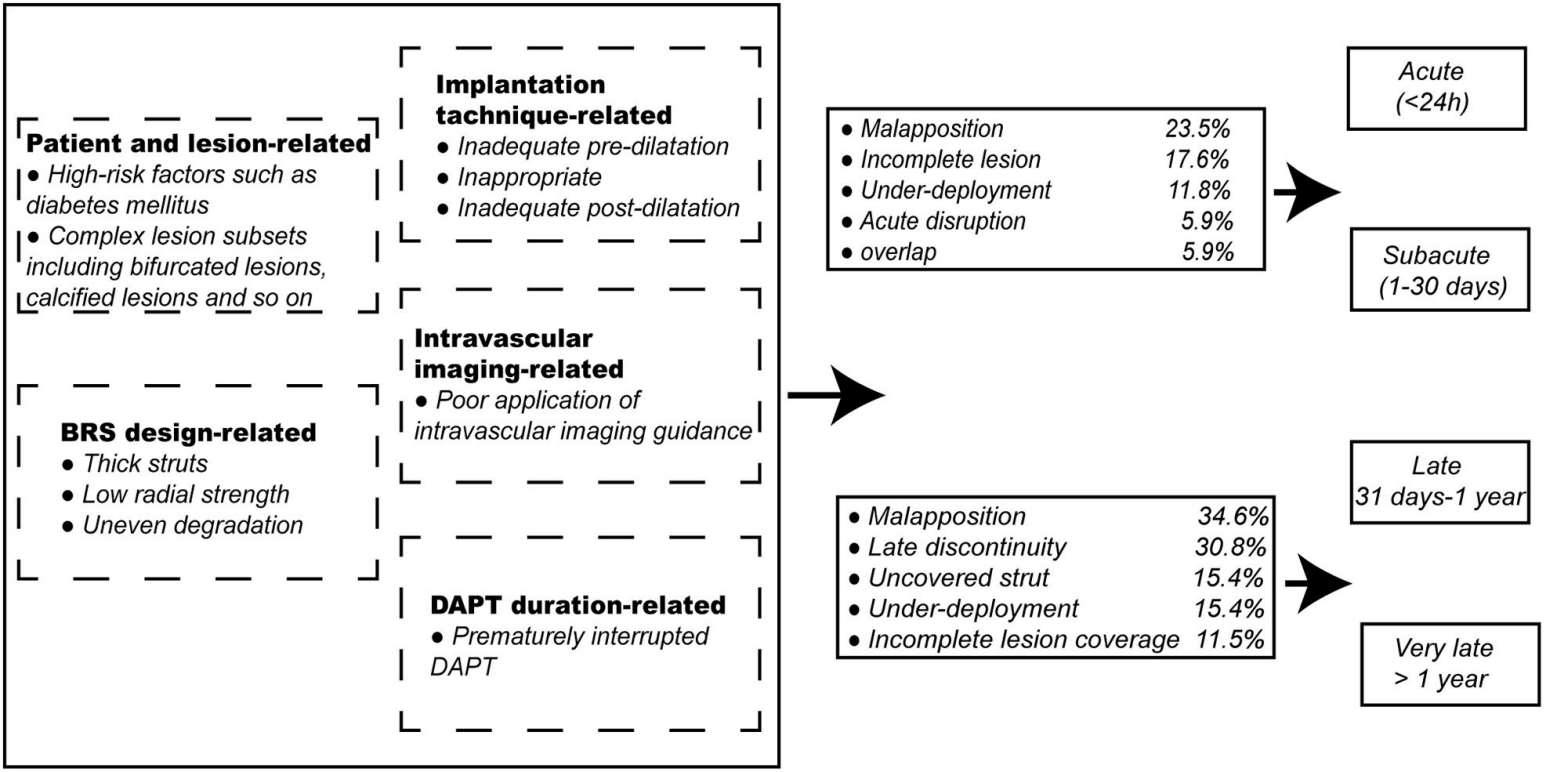
Related factors of scaffold thrombosis and the incidence of the representative imaging manifestations regarding scaffold thrombosis in acute/subacute and late/very late phases. BRS, bioresorbable scaffolds; DAPT, dual antiplatelet therapy.

Magmaris was believed to be associated with lower thrombogenicity, which was based mainly on the evidence that no ScT was observed after the Magmaris implantation in the BIOSOLVE-II and BIOSOLVE-III trials; thus far, however, ScT (0.3%) occurred only after the interruption of DAPT in the BIOSOLVE-IV trial (23, 24). The clinical outcomes with DREAM BRS in a series of BIOSOLVE trials initially looked favorable. Of note, caution is suggested in interpreting the results of these fairly small trials. In addition, Absorb BVS, the most well-studied BRS worldwide, was associated with a higher risk of ScT than contemporary DES (34). A meta-analysis of 25 studies including 10,510 patients showed that the risk of ScT significantly increased among patients with the Absorb BVS (odds ratio [OR] 2.06, 95% CI 1.07 to 3.98, *p* = 0.03), and the rates of definite ScT and ScT at 1 month were numerically higher as well (OR 1.91, 95% CI 0.82 to 4.46, *p* = 0.13, and OR 2.02, 95% CI 0.69 to 5.93, *p* = 0.20, respectively) (35). A meta-analysis of data from the seven ABSORB trials demonstrated that subacute, late, and very late ScT (VLScT) are significantly more common with the Absorb BVS than with the CoCr-EES, whereas no significant differences were found in the incidence of acute scaffold thrombosis (36). Similarly, another large-scale meta-analysis of 24 studies comprising data for 22,373 patients randomized assigned to BRS (*n* = 2,567) and EES (*n* = 19,806) revealed the 2 year outcomes after implantation. In the seven comparative studies, the risk for ScT at 2 years was significantly higher in BRS than in EES (OR 2.08, 95% CI 1.02 to 4.26), and the risk of VLScT tended to increase between 1 and 2 years (OR 2.03, 95% CI 0.62 to 6.71). In the 24 studies, the pooled estimated risk of ScT and VLScT was higher in BRS than in EES through 2 years (0.240 [95% CI 0.022 to 0.608]% vs. 0.003 [95% CI 0.000 to 0.028]%, and 1.43 [95% CI 0.67 to 2.41]% vs. 0.56 [95% CI 0.43 to 0.70]%, respectively) (37). Due to the high incidence of fatal complications such as ScT after Absorb BVS implantation, the clinical promotion and application of the device were limited and Absorb BVS was ultimately withdrawn from the global market on September 14, 2017. Herein, we analyze the causes of ScT and discuss how improvements can overcome this serious drawback. In addition, the incidences of the representative imaging manifestations regarding scaffold thrombosis in the acute/subacute and late/very late phases have been listed as well (Figure 2).

### Considerations on Patient and Lesion Characteristics

#### Patient Related

Numerous patient-related factors involved in ST of DES have been reported. The clinical factors linked with ST vary among the different time periods. For early ST, MI and heart failure seem to be strong risk predictors (38). Cardiogenic shock is a particularly predisposing condition because it combines insufficient coronary perfusion, liver dysfunction and limited drug absorption (39). For late ST, diabetes mellitus and renal failure are predisposing conditions (38), and the strongest predictor of late ST is a history of malignancy (OR 17.5, 95% CI 4.7 to 65.3) because of a prothrombotic phenotype and a high risk of bleeding under DAPT treatment (40). Because these predictors affect the overall state of the patient, confirmation of a similar association with ScT after BRS implantation can be expected.

#### Lesion Related

ST formation is more frequent in complex lesion subsets after DES implantation. A study demonstrated that lesion complexity was a significant independent correlate of early ST (adjusted OR 2.33, 95% CI 1.40 to 3.89) (41). Similarly, the challenge of complex lesions remains with BRSs and may even be amplified.

According to the current clinical experience, implanting vessels with reference vessel diameters of <2.25 mm and >3.75 mm is not recommended. Because BRSs tend to have thicker and wider scaffolds, the risk of ScT is increased after BRS implantation in very small vessels (<2.25 mm) (42). Moreover, we should pay attention to avoiding scaffold dislocation in regard to BRS implantation in large diameter vessels.

Implanting BRS in bifurcated lesions with already increased risk of ScT is a tremendous challenge. A sub study from the GHOST-EU registry showed that the rate of ScT (2.5%) with BRSs was higher than that with contemporary DES in bifurcation lesions. It is suggested that the operator made the decision on BRS size according to the proximal or distal reference diameter of the individual patient. BRS implantation in the main branch was performed before the proximal optimization technique. Side branch side opening, final proximal optimization technique and proximal optimization technique with mini-final kissing balloon dilatation are recommended when needed (43). In addition, due to the bulky design of BRSs, it is difficult to implant BRSs into side branches through main branch-BRS struts. A conventional DES is generally the first choice for side branch treatment (44).

Calcified lesions often lead to inadequate dilatation, and under expansion is an important contributory factor leading to ScT. Based on current clinical experience, calcification is not one of the contraindications for BRS implantation. Panoulas et al. have now proven that it is allowable to implant BRSs in adequately prepared calcified lesions and indicated that the cumulative 14 month rates of MACE were similar in non-calcified lesions (12.9% vs. 10.9%, log-rank *p* = 0.546) (45). However, implantation is still not recommended for heavily calcified lesions that are unable to be properly dilated.

Ostial lesions represent a challenging subset in the field of interventional cardiology due to concerns for acute recoil and restenosis. The GHOST-EU registry showed that treatment of coronary ostial lesions was an independent predictor of device-oriented composite endpoint (*p* = 0.0025, HR 2.65 [1.41 to 4.97]). The risk of ScT at 12 months was significantly higher than that in nonostial lesions (4.9% vs. 2.0%, log-rank *p* = 0.005). Notably, the unsatisfactory incidence of ScT in ostial lesions might be triggered by a low rate of intravascular guidance (32%) and post dilation (43%), which could contribute to malapposition and scaffold under expansion (46).

At present, the clinical application of BRSs is mostly limited to simple lesions, and data about the performance of BRS are generally blocked among the high-risk population. This situation also leads to a lack of clear recommendations on methodological approaches for complex lesions. Future clinical trials should have minimum exclusion criteria for evaluating the safety and effectiveness of BRSs and gaining more comprehensive clinical experiences.

### Considerations on BRS Design

BRSs generally have thicker struts than contemporary DES, which will increase the endothelial cell coverage area and migration distance and hinder the process of endothelialization. At the same time, the protrusion of the scaffolds into the lumen easily cause local laminar flow loss, and the formation of oscillating shear stress areas will cause local shear stress reduction. On the one hand, the change in local shear stress of BRS-implanted blood vessels can promote platelet activation and induce a thrombus cascade around stent trabeculae (47). On the other hand, it is more difficult to reendothelialize because of endothelial dysfunction caused by changes in local shear stress. These factors contribute to the formation of acute or subacute ST. In addition, PLLA is a common material for BRS platforms, and its radial strength and tensile strength are weaker than those of metals. Low radial strength can lead to elastic retraction, and low tensile strength can increase the occurrence of structural damage in the acute stage, which limits the use of post-expansion technology and causes poor expansion of scaffolds. Moreover, the uneven degradation of the overall scaffold structure can destroy the stability of the stent. The residual scaffolds protruding into the lumen can alter shear stress and hemodynamics and accelerate the formation of VLScT. Consistently, with the extension of follow-up time, the risk of VLScT was gradually increased (48). The 3 year follow-up of the ABSORB Japan trial showed that intraluminal scaffold dismantling was suspected to have a causal relationship with VLScT (49). In addition, data from 38 cases of VLScT in 36 patients showed scaffold discontinuity in 42.1% of cases using OCT imaging (50). These results suggest that the destruction of the BRS scaffold structure resulting from uneven degradation might be a vital mechanical cause of VLScT.

In the future, we should select the best materials to reduce the thickness of scaffolds and minimize coronary flow disturbance; on the other hand, it will improve the mechanical properties of scaffolds to ensure full adaptation to vascular anatomy. In addition, optimizing the time of stent degradation and strengthening the repair of the vascular intima will mitigate the damage caused by uneven degradation.

### Considerations on the Implantation Technique

A meta-analysis of the data from the 5 ABSORB prospective studies demonstrated that aggressive predilation with a ≥1:1 balloon-to-artery ratio and optimal postdilation were the independent predictors of freedom from ScT and TLF between 1 and 3 years (HR 0.44, *p* = 0.03, and HR 0.55, *p* = 0.05, respectively), which revealed that optimal implantation technique (predilation, vessel sizing, and postdilation) could improve clinical outcomes after BRS implantation (42).

#### Predilation

Before scaffolding, an appropriate size of non-compliant balloon should be used to predilatate with a ≥1:1 balloon-to-reference vessel diameter ratio to obtain residual 20 to 40% stenosis in 2 orthogonal views, which allows us to reduce the risk of thrombosis to a great extent. Additionally, the use of a scoring balloon contributes to facilitating BRS sizing and obtaining optimal scaffold expansion in complex lesions prior to BRS implantation (51).

#### Sizing

It is very important to select the appropriate scaffold size to match coronary artery lesions. Implantation of an undersized and presumably malapposed scaffold may influence the process of endothelial healing, and thus, the scaffold is at risk for collapsing into the lumen and dragging associated tissue, accelerating the formation of ScT. Moreover, compared with those in the non-oversized scaffold group, the 1 year MACE and MI rates were significantly higher in the group of implanted oversized scaffolds in a relatively small vessel (6.6% vs. 3.3%, log-rank *p* < 0.01, and 4.6% vs. 2.4%, log-rank *p* = 0.04), and a maximal lumen diameter smaller than the scaffold nominal size was one of the independent MACE determinants (OR 2.13, 95% CI 1.22 to 3.70, *p* < 0.01) (52). At the same time, it emphasized the importance of intravascular imaging in the process of stent implantation as well. The time of balloon expansion should also be extended to 30 s during stent deployment, which could fully expand BVS and allow us to obtain a larger luminal diameter. Due to frequent early recoil in fibrocalcific lesions, adequate stent deployment might be a vital part of the procedural technique for such lesions (53).

#### Postdilation

According to current practice recommendations, a non-compliant balloon should be inflated at 16–25 atm, with a nominal diameter up to 0.25–0.50 mm larger than that of the scaffold. Postdilatation leads to a result in which the residual stenosis is <10% in two orthogonal views and ensures that the entire scaffold adheres to the vessel wall. In the ABSORB II study, postdilatation was not recommended as a routine treatment, as it might increase the occurrence of underexpansion. A retrospective study showed that underexpansion (11.8%) was one of the common causes of ScT (54). In addition, the 3 year follow-up results of the ABSORB II study showed that the incidence of definite or probable ScT with Absorb BVS was significantly higher than that with CoCr-EES (3% vs. 0%, *p* = 0.0331) (55). Therefore, insufficient expansion during stent implantation might be an important reason for this unsatisfactory result (56). With the publication of the negative results of the ABSORB II study, the development of BRSs slowed considerably. Regardless, research findings from the ABSORB China trial are very encouraging. The 3 year clinical outcomes showed that the incidences of TLF and ScT after Absorb BVS implantation were similar to those in patients treated with CoCr-EES (5.5% vs. 4.7%, *p* = 0.68, and 0.9% vs. 0.0%, *p* = 0.50, respectively) (57). This is largely because the ABSORB China trial clarified the PSP principle (predilation, sizing and postdilation) and adhered to the principle throughout implantation (42).

### Considerations on Intravascular Imaging

Due to the higher scaffold thickness and greater recoil, BRS implantation may demand more accurate final optimization than contemporary DES, especially in complex lesions. However, under the detection of coronary angiography, BRSs had poor visibility, and the extent of stent degradation and vascular repair could not be accurately judged, which made it difficult to carry out the risk stratification of ScT and the optimization of an antithrombotic treatment strategy for the BRS-implanted population. Therefore, the application of intravascular imaging is of great value in the interventional treatment and follow-up of BRSs.

Accurate assessment of vascular size, plaque properties and lesion characteristics is a crucial step during BRS implantation. However, there is a tendency toward underestimation of vessel size with angiography compared with intravascular imaging (58). OCT is a unique tool for accurate measurement of vessel/scaffold sizing. A study suggested that OCT imaging guidance had a great influence in making decisions, with 16.4% of OCT pullbacks indicating the need for a different scaffold size (59). During the implantation process, intravascular imaging can help obtain the best vascular imaging results and detect immediate conditions such as the adherence of scaffolds, inadequate expansion and edge dissection. Optimization and timely posttreatment of BRS implantation will minimize the risk of scaffold underexpansion or malapposition and avoid scaffold rupture to reduce the occurrence of interventional complications and improve the clinical prognosis of patients. A retrospective analysis including 203 BRSs implanted in 101 consecutive patients found that OCT guidance significantly affected the procedural strategy during all phases of BRS implantation. Almost half of the frequency-domain OCT pullbacks (49%) indicated the need for changing strategies (59). Despite a default predilatation strategy adopted in most cases (98.8%) to achieve full lesion preparation with a 1:1 balloon/vessel ratio, OCT pullbacks led to further predilatation in 10.2% of cases (60). In addition, all patients underwent a postexpansion strategy under the guidance of aggressive systematic angiography, but 31.8% of lesions still needed additional expansion when OCT was used after BVS deployment, and OCT-guided postdilatation led to a minimal scaffold area increase of 0.8 mm^2^ (12.7%) and corrected malapposition in nearly one-third of lesions (59). Moreover, OCT follow-up after BRS implantation allows evaluation of scaffold degradation and vascular repair, which has important guiding significance for the formulation of individualized antithrombotic strategies.

Intravascular imaging provides unique application potential for the management of ScT. In the future, intravascular imaging will be widely used in the development and clinical application of BRSs, which has a profound impact and important practical value for preventing the occurrence of ScT.

### Considerations on DAPT Duration

DAPT plays an important role in the prevention of ST. Optimization of the time course of DAPT after stent implantation has been a controversial topic in the field of cardiovascular medicine. Clinicians must determine the best balance point to dodge the risk and gain the maximum benefits.

Current guidelines recommend that the duration of DAPT after DES implantation in patients with stable coronary artery diseases should be at least 6–12 months. For patients with acute coronary syndrome, a minimum of 12 months of DAPT is advised (61). Because scaffolds can gradually degrade over time, they avoid long-term residual implants in blood vessels and theoretically shorten the time course of DAPT. However, non-ideal clinical outcomes showed that the majority of these ScT events occurred in patients who prematurely interrupted DAPT after receiving BRS treatment. A meta-analysis including five randomized clinical trials (*n* = 1,730 patients) found that the rate of definite/probable device thrombosis was significantly higher in the Absorb BVS group than in the EES group (OR 2.93, 95% CI 1.37 to 6.26, *p* = 0.01). Of the 19 cases with definite/probable device thrombosis and known DAPT status in the Absorb BVS group, 37% (*n* = 7) were still receiving DAPT at the time of ScT, 47% were receiving aspirin monotherapy, and 16% had interrupted antiplatelet therapy. Similarly, in VLScT after Absorb BVS implantation, 92% (11/12) of the VLScT occurred in the absence of DAPT (62). Moreover, the INVEST Registry demonstrated that 83% of cases presenting VLScT were on single antiplatelet therapy, and only 17% were on DAPT at the time of events (50).

However, there is no large-scale prospective randomized trial to confirm whether a short duration of DAPT following BRS stenting will produce adverse effects. It is likely that the risk of thrombosis continues to lurk and is similar or even higher with BRSs than with the CoCr-EES owing to the still-present scaffolds. For this reason, a DAPT duration shorter than 12 months could not be recommended. Moreover, an observational study showed that among patients who were off DAPT, the incidence of ScT may have increased within the first 18 months post-implantation, with the highest incidence in the first month after DAPT termination (63). Thus, we recommend that patients receive a default 12 month DAPT regimen after BRS implantation. Prolonging the duration of DAPT even up to 3–4 years is a reasonable solution for patients with an increased ischemic risk and low bleeding risk because the stent is fully resorbed, and the vascular physiology is completely restored at 3–4 years after implantation. Nonetheless, extending DAPT beyond 1 year is not without risk because of the systemic effect of pharmacological platelet inhibition. This antithrombotic protection is achieved at the expense of an increased risk of bleeding. It is suggested that the decision about the duration of DAPT should be based on the individual patient rather than according to a one-size-fits-all principle. However, due to the limited data of clinical trials, it is urgent to carry out large-scale prospective randomized studies and registries to clarify the optimal duration of DAPT in patients undergoing BRS implantation.

## Conclusion

Overall, after the initial excessive enthusiasm stemming from the numerous attractive potential benefits of BRSs, the current results of clinical trials brought on a great disillusionment. BRSs seemed to be the darkness before the dawn. At present, DES devices remain the first choice in the majority of cases, but clinically available BRSs were given a class III indication outside of well-controlled clinical studies in the 2018 European Society of Cardiology (ESC) guidelines (64). There are still many limitations of BRSs; specifically, the higher-than-expected incidence of ScT represents the major bottleneck problem. Regardless, the disappointing outcomes mentioned above derive from the contribution of a variety of factors. In the future, it is highly likely that refined newer generation BRSs, with optimized implantation strategies and proper intravascular imaging, combined with long-term and effective DAPT regimens may shed new light on the application of BRSs.

## Author Contributions

XP designed and wrote the review and supervised and critically reviewed the complete manuscript. WQ, YJ, and YW performed the literature search and prepared the figures. BY and JT performed revisions and critically discussed the completed manuscript. All authors read and approved the final manuscript.

## Conflict of Interest

The authors declare that the research was conducted in the absence of any commercial or financial relationships that could be construed as a potential conflict of interest.

## Abbreviations

BMSs: Bare metal stents
BRSs: Bioresorbable scaffolds
CoCr: Cobalt-chromium
CoCr-EES: Cobalt-chromium everolimus-eluting stent
DAPT: Dual antiplatelet therapy
DES: Drug-eluting stents
ESC: European Society of Cardiology
IBS: Iron bioresorbable scaffold
IVUS: Intravascular ultrasonography
MACE: Major adverse cardiac effects
MI: Myocardial infarction
OCT: Optical coherence tomography
PCI: Percutaneous coronary intervention
PDLLA: Poly-d, l-lactide
PLGA: Poly-lactic-co-glycolic acid
PLLA: Poly-l-lactide
PTCA: Percutaneous transluminal coronary angioplasty
QCA: Quantitative coronary angiography
ScT: Scaffold thrombosis
ST: Stent thrombosis
TLF: Target lesion failure
TLR: Target lesion revascularization
VLScT: Very late scaffold thrombosis.
